# Conditional Depletion of Neurogenesis Inhibits Long-Term Recovery after Experimental Stroke in Mice

**DOI:** 10.1371/journal.pone.0038932

**Published:** 2012-06-19

**Authors:** Xiaomei Wang, XiaoOu Mao, Lin Xie, Fen Sun, David A. Greenberg, Kunlin Jin

**Affiliations:** 1 Buck Institute for Research on Aging, Novato, California, United States of America; 2 Department of Pharmacology and Neuroscience, University of North Texas Health Science Center at Fort Worth, Fort Worth, Texas, United States of America; 3 Institute for Aging and Alzheimer’s Disease Research, University of North Texas Health Science Center at Fort Worth, Fort Worth, Texas, United States of America; University of Nebraska Medical Center, United States of America

## Abstract

We reported previously that ablation of doublecortin (DCX)-immunopositive newborn neurons in mice worsens anatomical and functional outcome measured 1 day after experimental stroke, but whether this effect persists is unknown. We generated transgenic mice that express herpes simplex virus thymidine kinase under control of the DCX promoter (DCX-TK transgenic mice). DCX-expressing and recently divided cells in the rostral subventricular zone (SVZ) and hippocampus of DCX-TK transgenic mice, but not wild-type mice, were specifically depleted after ganciclovir (GCV) treatment for 14 days. Focal cerebral ischemia was induced by permanent distal middle cerebral artery occlusion (MCAO) on day 14 of vehicle or GCV treatment, and mice were killed 12 weeks after MCAO. Infarct volume was significantly increased and neurologic deficits were more severe in GCV- compared to vehicle-treated DCX-TK transgenic mice at first 8 weeks, after depletion of DCX- and bromodeoxyuridine-immunoreactive cells in the SVZ and dentate gyrus following focal ischemia. Our results indicate that endogenous neurogenesis in a critical period following experimental stroke influences the course of long-term recovery.

## Introduction

Stroke is the fourth leading cause of death in the United States, after heart disease, cancer, and chronic lung disease. Even in patients who survive stroke, 90% suffer permanent neurological deficits [1]. No effective treatment is available to reverse brain damage caused by stroke. Thus, stroke remains the leading cause of disability in the world. For many stroke survivors, the best hope is a lengthy program of rehabilitation, followed by life-long clinical support. However, even with rehabilitation therapy, 50% to 95% of stroke survivors remain impaired [2]. There is thus great need for new therapeutic developments in this area.

The finding that neuronal stem/progenitor cells (NSCs) persist in the rostral subventricular zone (SVZ) and the subgranular zone (SGZ) of the hippocampal dentate gyrus (DG) throughout life in mice [3], rats [4], non-human primates [5] and humans [6] suggests new therapeutic strategies for stroke, especially considering the increased proliferation of NSCs observed in the adult brain after injury. Focal cerebral ischemia stimulates NSC proliferation in the SVZ [4], and global ischemia has a similar effect in the dentate SGZ [7]. The resulting newborn neurons can migrate into the damaged brain regions [8], where they express phenotypic markers neuronal maturity (e.g., NeuN and MAP-2) [9], [10] and regional specificity (e.g., calbindin and dopamine- and cAMP-regulated phosphoprotein-32) [11], [12], and also form synapses [13].

Evidence for functional neuronal replacement has been reported in global cerebral ischemia, as intraventricular infusion of fibroblast growth factor-2 and epidermal growth factor promotes regeneration of hippocampal neurons, which integrate into existing circuitry and may help to ameliorate neurological deficits [9]. Others have employed cell-ablation techniques to demonstrate exacerbation of ischemic deficits, implying that the targeted cell population normally contributes to recovery. For example, whole-brain ionizing radiation, which ablates NSCs in the SGZ of guinea pigs [14] and mice [15], impaired performance on a water-maze task after global cerebral ischemia [14]. Irradiation of the immature brain, which also decreases hippocampal neurogenesis, increased infarct size and inflammation after hypoxic-ischemic brain injury in neonatal mice [15]. Cytosine-β-D-arabinofuranoside also inhibited SVZ neurogenesis after focal cerebral ischemia in adult rats [11], [16], although its anatomic and functional effects were not examined.

Ablation of NSCs by ionizing radiation and antimitotic drugs may also affect astrocytic, microglial, and endothelial cell lineages. To target NSCs more specifically, we generated transgenic mice that express herpes simplex virus-1 thymidine kinase (HSV-TK) under control of the promoter for doublecortin (DCX). HSV-TK can phosphorylate ganciclovir (GCV), a synthetic analogue of 2′-deoxy-guanosine, to GCV-monophosphate, which is further converted to GCV-diphosphate and GCV-triphosphate by host kinases. GCV-triphosphate causes premature DNA chain termination and apoptosis. In these (DCX-TK(+)) mice, immature neuronal (DCX-expressing) and recently divided (bromodeoxyuridine [BrdU]-labeled) cells in the SVZ and SGZ are specifically depleted after 14 days of GCV treatment. GCV-treated, DCX-TK(+) mice have larger infarcts and more severe sensorimotor behavioral deficits 1 day after stroke–induced by proximal middle cerebral artery occlusion (MCAO)–than do control mice [17]. This suggests that neurogenesis contributes to acute stroke outcome, but whether this effect persists in the long term, after neurogenesis is restored, is unclear.

In the current study, we examined the effect of NSC depletion on long-term anatomic and functional outcome from MCAO, using a less severe insult (distal MCAO) to ensure long-term survival. Our results indicate that acute postischemic neurogenesis exerts a persistent beneficial effect on outcome.

## Materials and Methods

### Generation of DCX-TK Transgenic Mice

Transgenic CD1 mice that express HSV-TK under control of the DCX promoter were generated at the Buck Institute for Research on Aging as described in our previous publication [17]. All animal procedures were conducted in accordance with National Institutes of Health guidelines and with the approval of the Institutional Animal Care and Use Committee of Buck Institute for Research on Aging.

### GCV Administration

Mice were anesthetized with 4% isoflurane in 70% N_2_O/30% O_2_, implanted with an osmotic minipump (Alzet 1003D), and infused continuously for 14 days with 0.25 µl/hr of either 20 mM GCV (Cytovene, Roche) or vehicle (PBS). MCAO was induced 14 days after the onset of GCV administration. Depletion of DCX-expressing cells was confirmed by immunohistochemistry.

### BrdU Administration

BrdU (50 mg/kg in saline; Sigma) was given by the intraperitoneal route twice daily for 24 hr before mice were euthanized (12 weeks post-MCAO). Brains were freshly isolated, and 50-µm coronal sections were cut with a cryostat and stored at −80°C. Some brains were perfused with 4% paraformaldehyde in PBS (pH 7.4) and embedded in paraffin.

### Permanent Focal Cerebral Ischemia

Male mice weighing 30–35 g were anesthetized with 2.0% isoflurane in 30% O_2_ and 70% N_2_O using a vaporizer. Distal MCAO was performed as previously described [18]. After making a 1 cm skin incision between the left eye and ear, a burr hole was drilled through the temporal bone. The dura mater was removed and the middle cerebral artery (MCA) was occluded permanently using a bipolar electrocoagulation forceps. Interruption of blood flow was confirmed under a microscope, and cerebral blood flow was measured by laser-Doppler flowmetry (Moor Instruments, Devon, England) in selected mice. During the operation, rectal temperature was maintained at 37±0.5°C with a thermostat-controlled heating blanket (Harvard Apparatus). After suturing the skin, mice were placed in a cage under an infrared heating lamp until recovery from anesthesia. Sham-operated mice underwent identical surgery except that the MCA was not occluded. Overall mortality in this MCAO model was <5%.

### Immunohistochemistry

Immunohistochemistry (5–6 animals per group) was performed as described previously [17]. Primary antibodies were mouse monoclonal anti-BrdU (2 µg/ml; Roche) and affinity-purified goat anti-DCX (1∶200; Santa Cruz Biotechnology); secondary antibodies were biotinylated donkey anti-goat or biotinylated horse anti-mouse IgG (both 1∶200; Santa Cruz Biotechnology). Sections were examined with a Nikon E800 epifluorescence microscope. Controls included omitting the primary and secondary antibodies.

### Dual-label Immunohistochemistry

Dual-label immunohistochemistry (5–6 animals per group) was performed as described elsewhere [17]. Primary antibodies were those listed above; secondary antibodies were Alexa Fluor 488-, 594-, or 647-conjugated donkey anti-mouse or anti-goat IgG (1∶200–500; Molecular Probes). Fluorescence signals were detected using an LSM 510 NLO Confocal Scanning System mounted on an Axiovert 200 inverted microscope (Carl Zeiss) equipped with a two-photon Chameleon laser (Coherent), and images were acquired using LSM 510 Imaging Software (Carl Zeiss). Two- or three-color images were scanned using Ar, 543 HeNe, 633 HeNe, and Chameleon lasers. Selected images were viewed at high magnification. Controls included omitting either the primary or secondary antibody or preabsorbing the primary antibody.

### Cell Counting

BrdU- and DCX-positive cells in SVZ and DG were counted in five to seven 50-µm coronal sections per animal (n = 6 per group), spaced 200 µm apart, by an observer blind to the experimental condition using a Zeiss microscope in bright field mode and a 40× objective. Two-photon confocal microscopy was used to count double-labeled cells. In SVZ, DCX- or BrdU-labeled cells were counted along the lateral walls of the lateral ventricles for a total of five to six sections per mouse, beginning 1.18 mm anterior to bregma. For the DG, all DCX- or BrdU-labeled cells within two cell diameters from the inner edge of the granule cell layer (GCL) of the dentate gyrus were included in the analysis. Results were expressed as the average number of BrdU- and DCX-positive cells in SVZ and DG per section.

### Histology

Mice (n = 5 each group) were anesthetized and decapitated 12 weeks after MCAO. Brains were removed and a series of adjacent 40-µm thick sections were cut in the coronal plane and then stained with hematoxylin and eosin (H&E). Contralateral and ipsilateral hemisphere areas were measured by a blinded observer using the NIH Image program, and areas were multiplied by the distance between sections to obtain the respective volumes. Volume loss (mm^3^) was calculated as a percentage of the volume of the structures in the control hemispheres according the following formula: [100×(V_C_−V_L_)/V_C_ (V_C_ = control hemisphere volume, V_L_ = lesioned hemisphere volume)], as described previously [19].

### Limb Placing Test

Limb placing (12 animals per group), which tests sensorimotor function [20], was evaluated bilaterally at 24 hr, 72 hr, 1 week, 2 weeks, 4 weeks, 8 weeks, and 12 weeksafter MCAO. Limb-placing tasks were scored by a blinded observer and forelimb and hindlimb scores based on the number of correct placing responses were averaged for each animal.

### Corner Test

The corner test (12 animals per group), which also assesses integrated sensorimotor function, was performed at 24 hr, 72 hr, 1 week, 2 weeks, 4 weeks, 8 weeks, and 12 weeks after MCAO as described [21], using two cardboard panels aligned to create a 30°corner. Twenty trials were performed for each mouse and the percentage of turns involving full rearing along either board was recorded. Normal mice rear equally to both sides whereas after MCAO, rearing to the unimpaired side predominates.

### Elevated Body Swing Test (EBST)

The elevated body swing test (12 animals per group) was used to evaluate symmetry of motor function, with the initial direction of upper body swing (>10°) recorded in three sets of 10 trials, performed over 5 min. The percentage of turns made to the side contralateral to the ischemic hemisphere (percent left-biased swing) was then calculated and average scores determined for each mouse. EBST was performed at 24 hr, 72 hr, 1 week, 2 weeks, 4 weeks, 8 weeks, and 12 weeks after MCAO.

### Beam-walking Test

The beam-walking test (12 animals per group) was performed to assess coordination and motor integration in the hindlimb [22] at 24 hr, 72 hr, 1 week, 2 weeks, 4 weeks, 8 weeks, and 12 weeks after MCAO as previously described with modification [22], [23], Each test session consisted of four trials (two in each direction), in which latency to cross a beam and the number of forelimb and hindlimb foot faults was recorded. Four trials were averaged to give a mean foot fault score.

### Statistical Analyses

Quantitative data were expressed as mean ± SEM from the indicated number of experiments. Behavioral data were analyzed by two-way analysis of variance (ANOVA) with repeated measures, followed by *post hoc* multiple comparison tests (Fisher PLSD or Student’s paired *t* test with the Bonferroni correction). Brain atrophy data were analyzed by one-way ANOVA followed by Fisher PLSD *post hoc* tests. *P* values <0.05 were considered significant.

## Results

**Figure 1:**
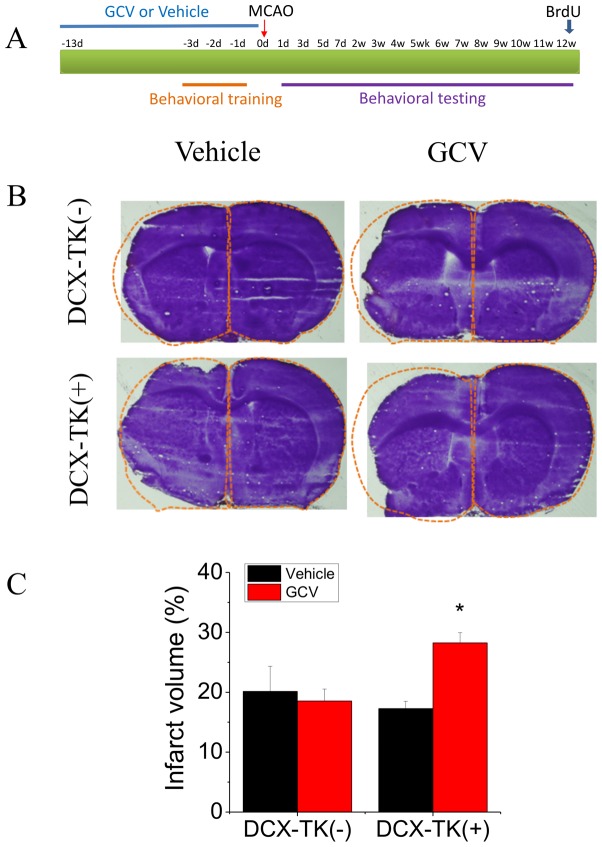
Brain volume loss in vehicle- and GCV-treated, wild-type and DCX-TK transgenic mice, 12 weeks after MCAO. (**A**) Transgenic (DCX-TK(+)) and wild type (DCX-TK(-)) mice were treated for 14 days with vehicle or GCV, received behavioral training, and then underwent MCAO. Behavioral testing was conducted for 12 weeks after MCAO, following which some mice were given BrdU for 1 day, and then all mice were euthanized for measurement of brain volume and immunohistochemistry. (**B**) H&E-stained coronal brain sections from vehicle- and GCV-treated DCX-TK transgenic (DCX-TK(+)) and wild-type **(**DCX-TK(-)) mice. Dashed lines delineate normal brain contour based on the nonischemic hemisphere. (**C**) Volume loss (expressed as a percentage of hemispheric volume) in vehicle (black)- and GCV (red)-treated DCX-TK(+) and DCX-TK (-) mice. *P<0.05 compared to vehicle-treated mice.

As shown in **Figure 1**, brain volume loss (atrophy) at 12 weeks post-MCAO was increased by ∼50% in GCV-treated DCX-TK(+) mice, compared to vehicle-treated DCX-TK(+) and either GCV- or vehicle-treated wild-type (DCX-TK(-)) mice.

**Figure 2:**
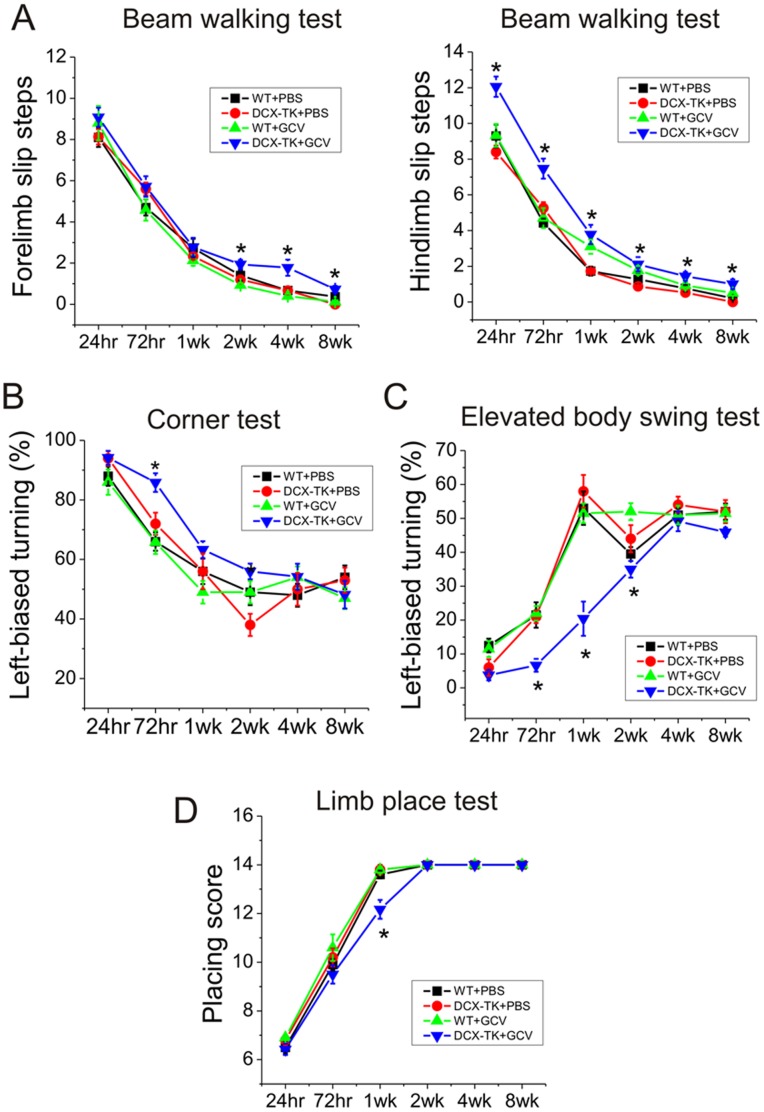
Neurobehavioral deficits in vehicle- and GCV-treated, wild-type and DCX-TK transgenic mice, 12 weeks after MCAO. Transgenic (DCX-TK(+)) and wild type (DCX-TK(-)) mice were treated for 14 days with vehicle (PBS) or GCV, then underwent MCAO. Behavioral testing was performed at the indicated times after MCAO. (**A**) Beam-walking test scores, expressed as the mean numbers of forelimb (left panel) or hindlimb (right panel) slip steps when traversing an elevated narrow beam; higher scores represent more severe deficits. (**B**) Corner test scores, expressed as a percentage of rearing to the contralesional (impaired) side; lower scores represent more severe deficits. (**C**) Elevated body swing test scores, expressed as a percentage of turns to the contralesional (impaired) side; lower scores represent more severe deficits. (**D**) Limb-placing test scores, expressed as a score derived from the number of correct limb placements; lower scores represent more severe deficits. *, *P*<0.05 compared to vehicle-treated DCX-TK(+) mice.

Neurobehavioral testing (n = 12 per group) was performed in the same four experimental groups at 24 hr, 72 hr, 1 week, 2 weeks, 4 weeks, 8 weeks, and 12 weeks post-MCAO (**Figure 2**). GCV-treated DCX-TK(+) mice performed worse than mice from the other three groups at one or more time points in each test. The beam walking test (especially testing for hindlimb slips) showed the most consistently deficient performance over the first 8 weeks post-ischemia; the elevated body swing test and corner test tended to show impairment early in the course, whereas a relative deficit in forelimb slip steps on the beam walking test was observed later. No differences between groups could be detected at the 12-week time point (not shown).

**Figure 3:**
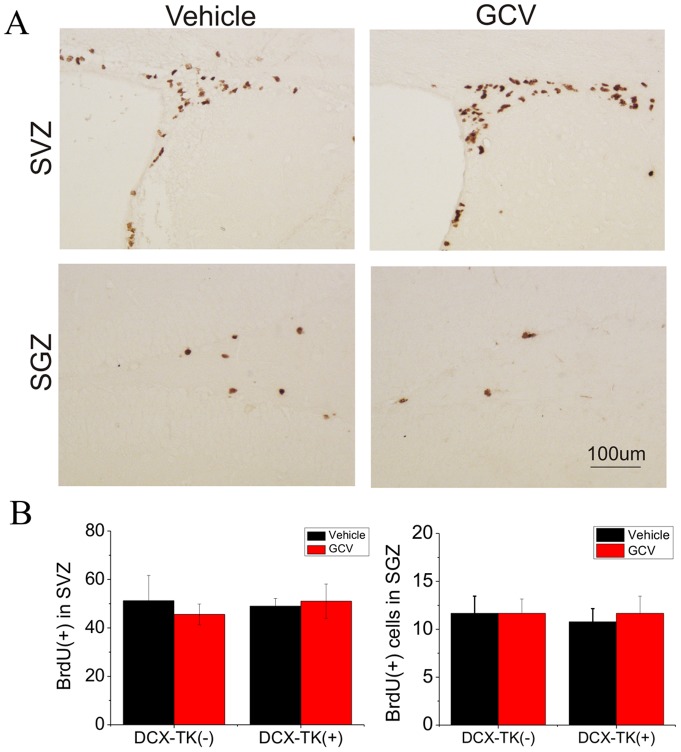
BrdU-immunopositive cells in SVZ and dentate SGZ of vehicle- and GCV-treated, wild-type and DCX-TK transgenic mice 12, weeks after MCAO. Mice were treated as described in the legend to Figure 1. (A) Representative images of BrdU-immunoreactive cells in SVZ (top) and dentate SGZ (bottom) from vehicle (left)- and GCV (right)-treated DCX-TK(+) transgenic mice. (B) Quantification of BrdU-immunoreactive cells in SVZ (left panel) and dentate SGZ (right panel) from GCV (red bars)- and vehicle (black bars)-treated DCX-TK(+) and DCX-TK(-) mice. There were no significant differences between vehicle- and GCV-treated groups.

**Figure 4:**
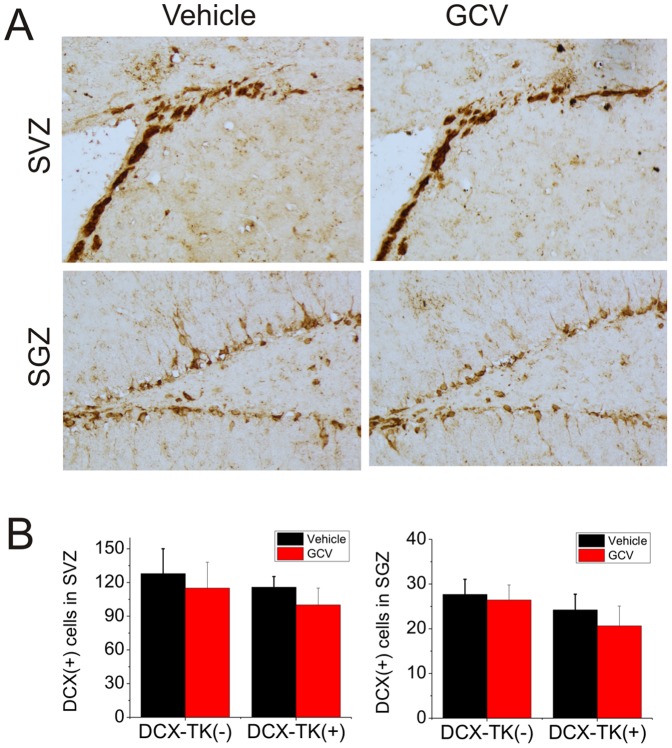
DCX-immunopositive cells in SVZ and dentate SGZ of vehicle- and GCV-treated, wild-type and DCX-TK transgenic mice, 12 weeks after MCAO. (A) Representative images of DCX-immunoreactive cells in SVZ (top) and dentate SGZ (bottom) from vehicle (left)- and GCV (right)-treated DCX-TK(+) transgenic mice. (B) Quantification of DCX-immunoreactive cells in SVZ (left panel) and dentate SGZ (right panel) from GCV (red bars)- and vehicle (black bars)-treated DCX-TK(+) and DCX-TK(-) mice. There were no significant differences between vehicle- and GCV-treated groups.

**Figure 5:**
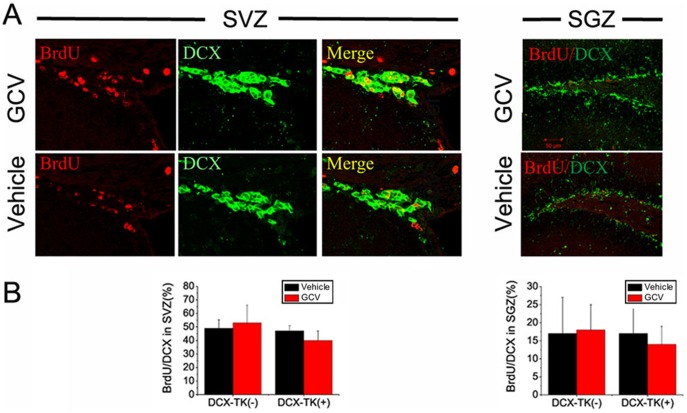
BrdU/DCX dual-immunopositive cells in SVZ and dentate SGZ of vehicle- and GCV-treated, wild type and DCX-TK transgenic mice, 12 weeks after MCAO. (A) Two-photon confocal images of BrdU (red)-, DCX (green)-, and BrdU/DCX-immunoreactive cells in SVZ (left panels) and SGZ (right panels) of GCV (top)- and vehicle (bottom)-treated DCX-TK(+) transgenic mice. (B) Quantification of BrdU/DCX dual-immunoreactive cells in SVZ (left panel) and SGZ (right panel) from GCV (red bars)- and vehicle (black bars)-treated DCX-TK(+) and DCX-TK(-) mice. There were no significant differences between vehicle- and GCV-treated groups.

Next we asked if numbers of BrdU- and DCX-immunopositive cells were restored during the 12-week postischemic interval, after administration of GCV was discontinued. **Figure 3** illustrates that when BrdU was administered for 1 day prior to euthanizing mice at 12 weeks, there was no significant difference in the number of BrdU-positive cells in the SVZ or SGZ among GCV- or vehicle-treated, DCX-TK(+) or DCX-TK(-) mice. As shown in **Figure 4**, the same was true when DCX-positive cells were counted. Thus, both the rate of cell division (BrdU incorporation over 24 hr) and the number of new neurons (DCX-immunopositive cells) no longer showed an effect of prior GCV treatment, suggesting that normal neurogenesis was restored. This time course is consistent with the observation that neurogenesis begins to return by 6 weeks after GCV treatment in nestin-TK transgenic mice (24) and reaches approximately half-normal levels by 8 weeks in the DCX-TK(+) mice we studied. Dual-label immunohistochemistry showed that ∼50% of DCX-positive cells in SVZ and ∼15% of DCX-positive cells in SGZ incorporated BrdU (**Figure 5**), reflecting the limited efficiency of BrdU labeling.

## Discussion

We reported previously that conditional depletion of DCX-expressing cells in neurogenic brain regions, achieved by administration of GCV to DCX-TK(+) transgenic mice, increased volume loss and neurobehavioral deficits measured 1 day after MCAO (17). This finding suggests that endogenous neurogenesis normally promotes a more favorable acute outcome after stroke, since its ablation worsens outcome, and studies in which NSCs were ablated by other means support a similar conclusion (14,15,24).

In the present study, we asked whether the adverse effect of transiently ablating NSCs is persistent. Volume loss remained greater in DCX-TK(+) than in control young-adult mice 12 weeks after MCAO, whereas neurobehavioral deficits, which were initially greater in DCX-TK(+) mice, gradually equalized. No significant neurobehavioral differences were found in GCV-treated DCX-TK(+) mice, compared to vehicle-treated DCX-TK(+) mice at 12 weeks after MCAO. Of interest, neurogenesis in SVZ and SGZ also returned to normal levels by 12 weeks. We conclude that ablation of endogenous neurogenesis exerts an early, persistent effect on damaged size, whereas its effect on neurobehavioral outcome is transient. This transiency may or may not be related to restoration of neurogenesis after GCV treatment is discontinued. These findings add further support to the notion that endogenous neurogenesis exerts a beneficial influence on stroke outcome.

The DCX-TK(+) transgenic mouse model that we used relies on the fact that GCV is phosphorylated by HSV-TK and ultimately incorporated into newly synthesized DNA. This results in the arrest of DNA synthesis and leads to subsequent DNA fragmentation [24]. Since HSV-TK is under the control of the DCX promoter, only dividing DCX-expressing cells are depleted. We chose to target this cell population to ablate neurogenesis because in adult brain, DCX is expressed almost exclusively in newborn and migrating neurons (26–28). We found previously that GCV treatment of DCX-TK(+) mice for 14 days depletes DCX-positive cells from SVZ and DGZ (17), which was the basis for adopting this treatment interval in the present study. We also observed that this treatment regimen does not deplete other cell types, notably astrocytes, and is not associated with microglial activation suggestive of an inflammatory response that could affect untargeted bystander cells (17).

One difference between this and our previous study is that, in the present case, the MCA was occluded distal to the origin of the lenticulostriate arteries, producing a cortical infarct, but sparing the striatum. This was done because long-term survival is better after distal than after proximal MCAO, and our goal was to study long-term persistence of the effects of NSC ablation. We have shown that both corticostriatal infarcts (4) and infarcts involving only cortex (29) enhance endogenous neurogenesis. Although the present study suggests that endogenous neurogenesis promotes improved outcome after MCAO, it does not address the mechanism involved. After MCAO, some newborn neurons migrate into the ischemic striatum and cerebral cortex (8,11,12,30), and a subpopulation of these assume phenotypic features of mature neurons (31), including tetrodotoxin-sensitive Na^+^ action potentials and spontaneous excitatory post-synaptic currents (32), suggesting that NSCs are able to reestablish local interneuronal connections and synaptic connectivity ischemia [25], and neuronal replacement may be one of mechanisms underlying neurogenesis-mediated beneficial effect in the chronic phase after stroke. Notably, only a small portion of SVZ-derived cells differentiate into functional mature neurons, and most newborn cells in the SVZ appear to die during migration after focal ischemia [11], perhaps due to local hypoxic environment, which prevent from cell survival. Therefore, other mechanisms may be also involved in neurogenesis-mediated functional recovery in the late stage of stroke, which include, but not limit to, that NSCs act as local pumps to release the neurotrophic and growth factors, such as brain-derived neurotrophic factor (BDNF), glial derived neurotrophic factor (GDNF) and never growth factor (NGF), etc. Those factors, in turn, support cell function and prevent cascade of apoptosis or further prevents subsequent cell death [26].

If endogenous neurogenesis contributes to a more favorable outcome from stroke, as this and other (14,15,17,24) studies suggest, therapeutic implications might follow. Clinically used drugs such as antidepressants and mood stabilizers, as well as environmental enrichment and physical activity, can all enhance endogenous neurogenesis. This property could be exploitable in the clinical management of stroke.
